# Supplementary material for: HNF1A POU Domain Mutations Found in Japanese Liver Cancer Patients Cause Downregulation of HNF4A Promoter Activity with Possible Disruption in Transcription Networks
Source: Genes (Basel). 2022 Feb 24;13(3):413. doi: 10.3390/genes13030413 (PMC8949677; doi:10.3390/genes13030413)

Table S1: Average values and standard deviations of RMSD, fluctuation, and SASA for each run shown in Figure 3E. The standard deviations are provided in parentheses. The average values and standard error of the 10 runs are presented in the rightmost column

	run1	run2	run3	run4	run5	run6	run7	run8	run9	run10	ave. and std. err. among 10 runs
RMSD of WT protein	2.5(0.1)	3.2(0.6)	2.1(0.5)	2.8(0.4)	1.9(0.2)	2.7(0.4)	1.9(0.2)	1.7(0.3)	1.8(0.2)	1.9(0.2)	2.2(0.2)
RMSD of mutant protein	2.6(0.2)	2.4(0.3)	2.0(0.2)	3.1(0.8)	2.1(0.3)	1.9(0.2)	2.1(0.2)	2.5(0.6)	2.5(0.6)	1.9(0.3)	2.3(0.1)
Fluct. of WT N-ter.	3.4(1.0)	3.7(1.6)	3.9(1.4)	3.8(1.1)	2.8(1.0)	3.6(1.5)	3.6(0.7)	2.6(0.7)	3.1(0.9)	3.9(0.8)	3.4(0.1)
Fluct. of mutant N-ter.	6.8(3.5)	7.1(3.9)	2.8(1.0)	7.7(3.7)	4.4(1.6)	7.3(1.4)	3.7(1.1)	4.8(0.7)	4.4(1.6)	10.3(1.3)	5.9(0.7)
SASA of WT 259th residue	0.3(0.6)	0.4(0.7)	2.1(2.6)	0.8(1.4)	1.8(1.6)	1.1(1.1)	0.8(1.6)	0.4(0.7)	1.3(1.7)	0.5(1.0)	0.9(0.2)
SASA of mutant 259th residue	6.9(6.0)	19.9(14.1)	6.1(4.6)	18.7(18.5)	4.8(4.9)	2.4(3.1)	0.9(1.6)	4.2(3.9)	3.0(2.9)	6.8(7.9)	7.4(2.0)

Table S2: List of Primer and siRNA sequences used in the study

Gene Name	Primer Sequence (5' to 3')	Species	Purpose
HNF1A	5'-gggcggccgcggtttctaagctgagccagc-3'	Mouse	Cloning Primers
HNF1A	5'-ggggtaccttactgggaagaggaggcc-3'	Mouse	Cloning Primers
Y122C HNF1A Mutant	5'-tgttgtgctgctgcaagcacgacttgaccatcttc-3'<br>5'-gaagatggtcaagtcgtgcttgcagcagcacaaca-3'	Mouse	Mutagenesis
R229Q HNF1A Mutant	5'-tccaccaaggtctcttgctcttccttgctgg-3'<br>5'-ccagcaaggaagagcaagagaccttggtgga-3'	Mouse	Mutagenesis
V259F HNF1A Mutant	5'-acctccgtgaaaaggttggagcctagccc-3'<br>5'-gggctaggctccaaccttttcacggaggt-3'	Mouse	Mutagenesis
Y122C HNF1A Mutant	5'-ttgtgctgctgcaggcaggacttgaccatcttc-3'<br>5'-gaagatggtcaagtcctgcctgcagcagcacaa-3'	Human	Mutagenesis
V259F HNF1A Mutant	5'-cgcacctccgtgaagaggttggagccc-3'<br>5'-gggctccaacctcttcacggaggtgcg-3'	Human	Mutagenesis
HNF4A P2-2200 (Containing HNF1A Binding site GTTACTCTTTAAC)	5'-ccctaagtgactggttactctttaacgtatccacccacc-3'<br>5'-ggtgggtggatacgttaaagagtaaccagtcacttaggg-3'		EMSA
Reverse Primer hGH poly A terminator	5'-gcactggggaggggtcacag-3'		Flanking Primers
GAPDH	5'-ggagcgagatccctccaaaat-3'<br>5'-ggctgttgtcatacttctcatgg-3'	Human	
siRNA HNF4A 1	S- GAC AUU CGG GCG AAG AAG AdTdT<br>A- UCU UCU UCG CCC GAA UGU CdGdC		
siRNA HNF4A 2	S- CAC AAU GCC CAC UCA CdTdT<br>A- GUG AGU GGG CAU UGU GdTdT		

Figure S1. The ability of the (A,C) human WT and mutant HNF1A and (B,D) mouse WT and mutant HNF1A to transactivate the target promoter (A,B—*HNF4A*-P2; C,D—*HNF4A*-P2-2200) when overexpressed in HEK293 cells. (E) The ability of the human WT and mutant HNF1A to transactivate the target promoter (*HNF4A*-P2-2200) when overexpressed in Huh7 cells. The cells were co-transfected with the indicated luciferase reporters, and either an empty expression vector (serving as a control) or expression vectors (100 ng) for the indicated HNF1A proteins in 24-well culture plates. The bars indicate the fold activation for HNF1A WT and mutants (vs. control) for target promoters. The corresponding promoter activity is reported as fold activation over control (±SEM, $n = 3$). The data reported represent the averages of three experiments, each performed in duplicate. (*, $p < 0.05$; **, $p < 0.01$; ***, $p < 0.001$).

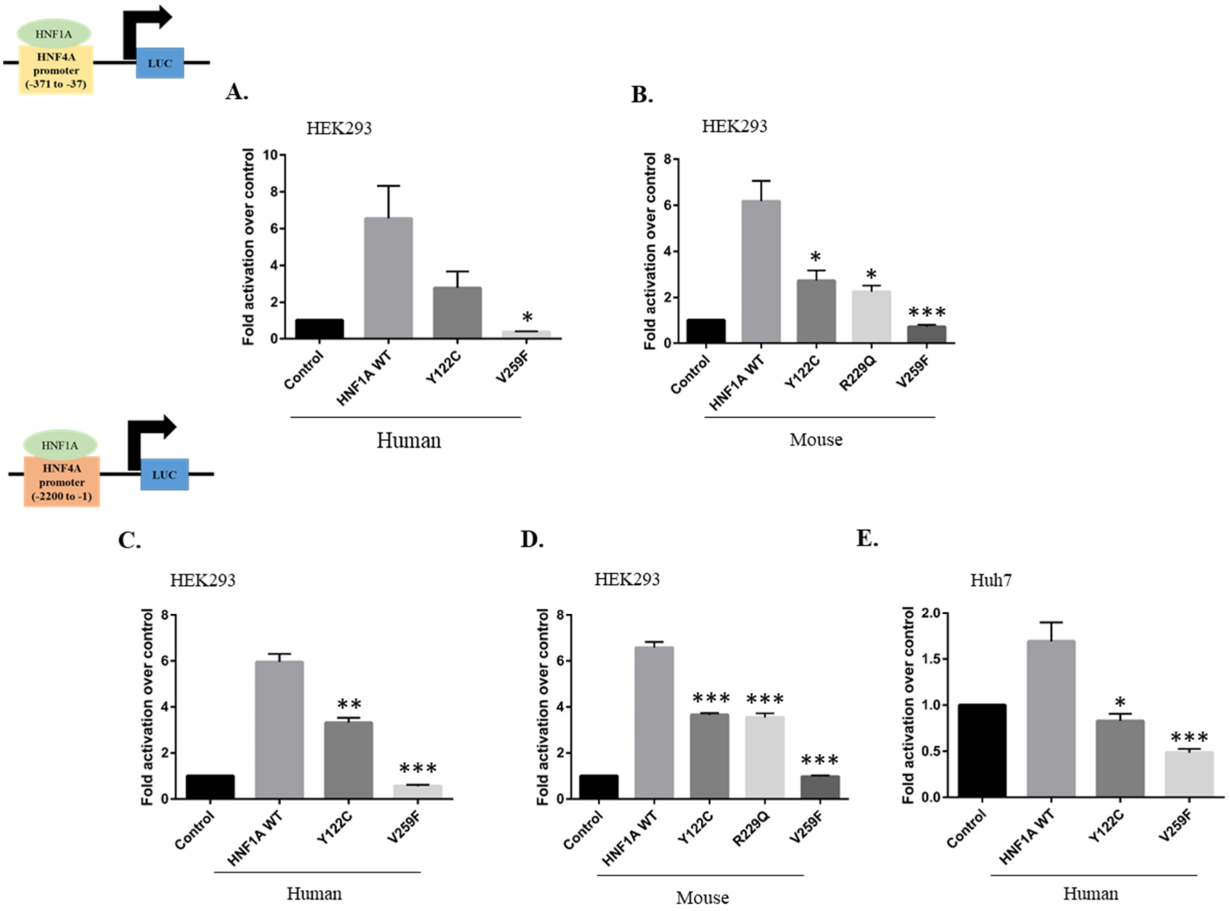

Figure S2. HEK293 cells were transfected with expression vectors encoding HNF4A WT or the indicated mutants. (A) Western blot analysis shows that all proteins were similarly expressed. (B) EMSA analysis was used to assess the binding of WT or mutated HNF4A nuclear proteins to a double-stranded oligonucleotide corresponding to the consensus HNF4A-binding elements of the *HNF1A* and *ApoB* promoter region. (C) Structural simulation analysis of the RMSF revealed that mutants have a higher fluctuation rate than WT HNF4A.

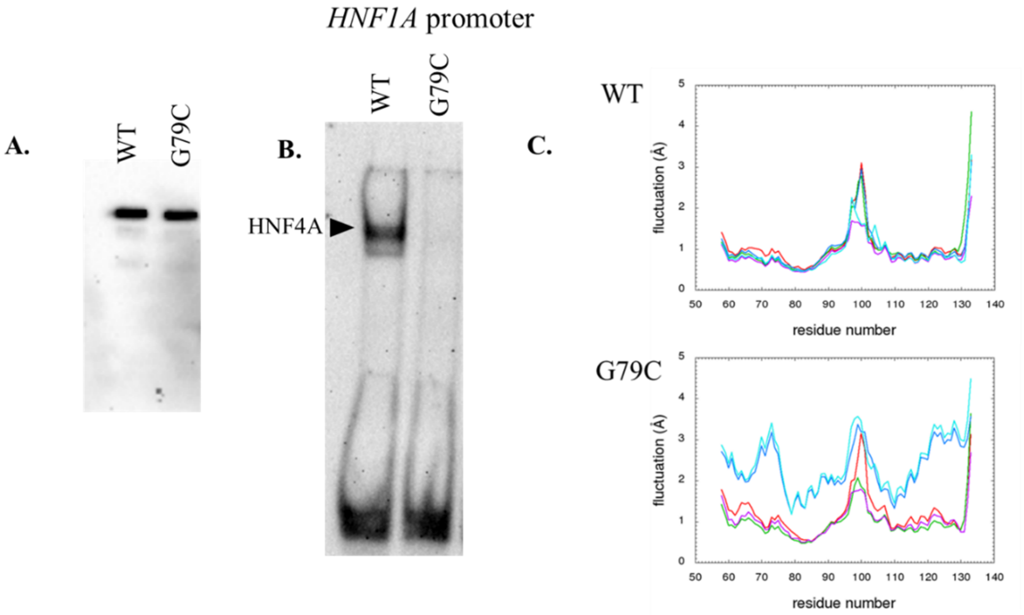

Figure S3. HNF1A mRNA expression is strongly associated with HNF4A mRNA expression in different cancers. RNA-sequencing data from the TCGA database revealed that HNF4A mRNA expression is highly correlated with HNF1A expression. HNF4A vs. HNF1A. The correlation of gene expression between HNF1A and HNF4A genes was tested using the Spearman's rank correlation test. A positive correlation between HNF1A expression and HNF4A levels was found in CHOL (r = 0.28, *p* = 0.066), COAD (r = 0.52, *p* = 1.1 × 10^{-23}), KIRC (r = 0.64, *p* = 1 × 10^{-70}), KIRP (r = 0.77, *p* = 7.3 × 10^{-65}), LIHC (r = 0.53, *p* = 4.6 × 10^{-31}), LUAD (r = 0.56, *p* = 4 × 10^{-45}), PAAD (r = 0.71, *p* = 7.8 × 10^{-29}), STAD (r = 0.75, *p* = 5.7 × 10^{-82}) and READ (r = 0.48, *p* = 3.4 × 10^{-7}; Supplementary File 1, Figure S3). CHOL—cholangiocarcinoma; COAD—colon adenocarcinoma; KIRC—kidney renal clear cell carcinoma; KIRP—kidney renal papillary cell carcinoma; LIHC—liver hepatocellular carcinoma; LUAD—lung adenocarcinoma; PAAD—pancreatic adenocarcinoma; STAD—stomach adenocarcinoma; READ—rectal adenocarcinoma.

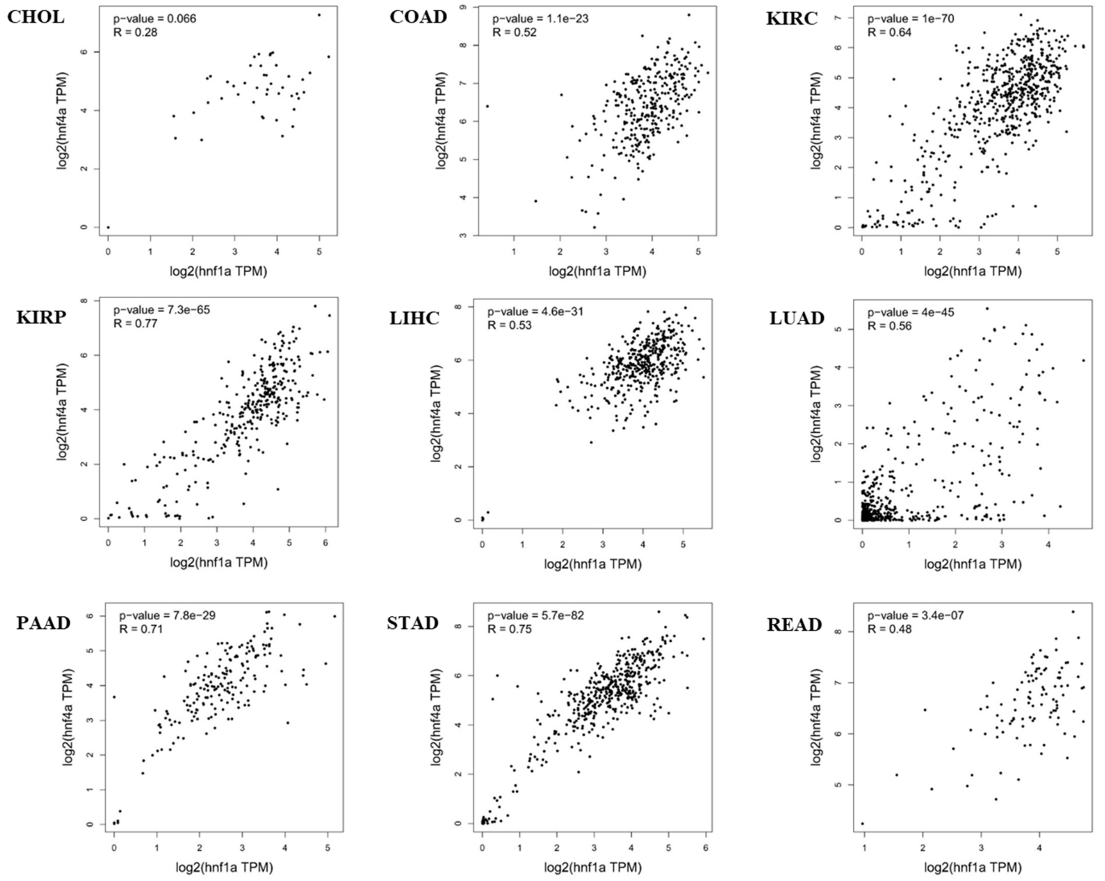

Figure S4. Gene ontology analysis of top 10 molecular functions.

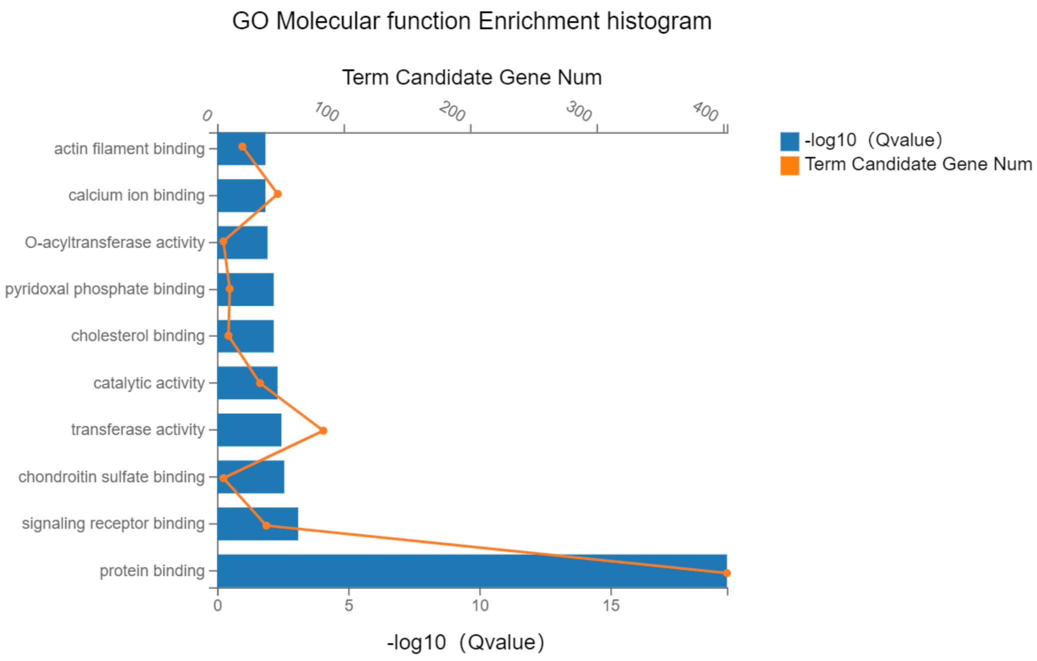

Supplement: Supplementary file 1 [file genes-13-00413-s001.zip › Supplementary File S1.pdf]
